# Challenges in policy reforms for non-communicable diseases: the case of diabetes in Kenya

**DOI:** 10.1080/16549716.2019.1611243

**Published:** 2019-05-23

**Authors:** Veronica Shiroya, Florian Neuhann, Olaf Müller, Andreas Deckert

**Affiliations:** a Department of Disease Control in Disadvantaged Populations, Heidelberg Institute of Global Health, Medical Faculty of Ruprecht-Karls-University Heidelberg, Heidelberg, Germany; b Department of Epidemiology and Biostatistics, Health Promotion Alliance of Kenya, Kitale, Kenya

**Keywords:** Health policy, LMIC, qualitative study, implementation, monitoring, citizen involvement, prevention, population-based

## Abstract

**Background**: The 2011 UN declaration on non-communicable diseases (NCDs) recognized their importance as a global health issue, particularly for low- and middle-income countries. The extent to which these countries address policy implementation gaps in the face of resource limitations and competing priorities remains largely unexplored.

**Objective**: This qualitative study presents Kenya’s experience of translating the UN declaration to national policies for diabetes prevention and control.

**Methods**: Policy documents published between 2006 and 2016 were analyzed. Thirty-two documents were included in the analysis. Interviews with six purposively selected policy stakeholders at multiple levels of decision-making were conducted. Emerging themes were deconstructed into a policy analysis triangle.

**Results**: Diabetes-specific policies already existed in Kenya before 2011, suggesting successful advocacy work by diabetes interest groups. The 2011 UN declaration subsequently coincided with a period of political transition in Kenya, opening policy windows that the diabetes community leveraged to trigger political drive against prevailing challenges. The post-declaration period reflected a transition from diabetes-specific policies to a wider NCD agenda. Most of the documents and national strategies aligned strongly with international documents, however, were based on scant local evidence. The implementation process was largely health-sector driven. The non-health sector remained largely uninvolved, contrary to global recommendations. This, in addition to fragmented health governance and weak monitoring systems, continues to undermine existing gains and efforts to fight diabetes and NCDs on a wider scale.

**Conclusions**: In Kenya, a major gap remains between how diabetes is addressed within the NCD policy agenda and tackling diabetes in reality, with respect to local implementation processes. More emphasis on population-wide prevention and inclusion of the non-health sector could help to cascade national efforts to the grassroots level. A concerted effort from the highest political level is needed to address overarching NCD drivers while maintaining health system improvement strategies.

## Background

Similar to many resource-limited countries in Sub-Saharan Africa (SSA), Kenya recognizes a growing burden of non-communicable diseases (NCDs) that challenges the health system [1]. Specific problems of NCDs among the global poorest billion are addressed in the Lancet NCDs and Injuries Poverty Commission, which includes Kenya as one of its member countries [2]. Kenya’s Ministry of Health predicts that NCDs will be the country’s main disease burden by 2027 [3]. Currently, NCDs account for more than half (50–70%) of hospital-bed occupancy rates and are responsible for up to 50% of inpatient deaths [3,4]. This illustrates a serious challenge for the health system due to the double burden of persisting infectious diseases and the additional threat due to NCDs. The continuing issue of poverty as a social determinant of health compounds further on this burden.

For convenience only, we use ‘diabetes’ to refer to diabetes mellitus type 2 in the following and throughout the article; diabetes mellitus type 1 is not addressed here. The NCD diabetes is an important contributor to global morbidity and mortality [5,6]. In Kenya, diabetes prevalence was between 2.7% (rural) and 10% (urban) in 2012 [7,8]. In the same year, for impaired glucose tolerance the rates were estimated at 8.8% (rural) and 14.4% (urban) [9]. In a region (SSA) estimated to have the highest number of undiagnosed people living with diabetes [10,11], it is likely that these figures from Kenya are underestimated. Moreover, an increasing proportion of young people is diagnosed with diabetes in Kenya, where greater than 59% of the population is under 25 years of age [9,12,13]. In 2015, only 41% of Kenyans aged 15–69 and diagnosed with diabetes received treatment and only 7% of them had controlled the disease [14]. This pattern reflects a phenomenon already described as the ‘rules of halves’ where more diagnostic efforts yield more cases, with many of them not treated accordingly [15]. Urbanization and lifestyle changes, e.g. increased consumption of refined foods and physical inactivity, are some factors contributing to this trend as Kenya transitions economically, epidemiologically and demographically [12,16]. One-quarter of children in Kenya are stunted and starvation in utero and early life has been associated with the development of diabetes in adulthood [17–22]. These trends suggest that heightened and sustained life-course approaches to prevent NCDs at the population level are necessary.

Next to prevention, there is a need for NCD care improvement. A study in Kenya’s largest referral hospital found 30% of patients with diabetic ketoacidosis died within 48 h after presentation, accounting for 8% of diabetic admissions [8,16] Such late presentation for care, alongside challenges like shortage of health-care workers and limited knowledge on how to manage diabetes; high cost of insulin and inadequate patient follow-up compound the dilemma for patients [8,23]. A national health facility census in 2013 and a health system assessment in 2017 further exemplified that Kenyan health facilities are largely unprepared to provide NCD services [24,25].

The 2011 UN Summit provided a global platform for NCDs, although national governments and actors were left to ‘customize the implementation’ of their commitments [26–28]. A global monitoring framework with voluntary targets was adopted to follow global progress on NCDs [6]. Civil society groups under the umbrella of the International Diabetes Federation (IDF) have developed scorecards to track progress on the IDF Global Action Plan on diabetes 2011–2021 [10,29]. Such approaches through citizen monitoring (active involvement of patients and their communities) and shadow reporting (alternative reports by civil society groups) as in HIV/AIDS could be important elements for providing balance and objectivity to scrutinize governmental progress reports.

This paper explores how the Kenyan policies address diabetes as a marker condition for NCDs, focusing on the question, ‘How did Kenya translate the global UN declaration on NCDs to local action for national policy reforms towards diabetes prevention and control?’.

## Methods

### Study design

This study, which was conducted between March 2016 and June 2017, explored the policy environment impacting diabetes prevention and control in Kenya. Health policy in this paper is understood as ‘the decisions, plans, and courses of actions (and inactions) for diabetes control taken by a set of institutions and organizations – national, state and local – to advance the public’s health’ [30,31]. The work steps included a desk review of policy documents, followed by interviews with key informants at multiple levels of NCD policy implementation.

The objectives were to:

1. Identify existing diabetes and/or NCD policies up to 2016 (year of data collection), the components of the policy development process, and the actors involved.
2. Summarize the post-UN NCD declaration implementation of existing diabetes policies and interventions on different political levels.
3. Identify challenges and gaps in diabetes prevention and control.
4. Highlight how some implementation challenges are being addressed.

### Document review

**Figure 1.**
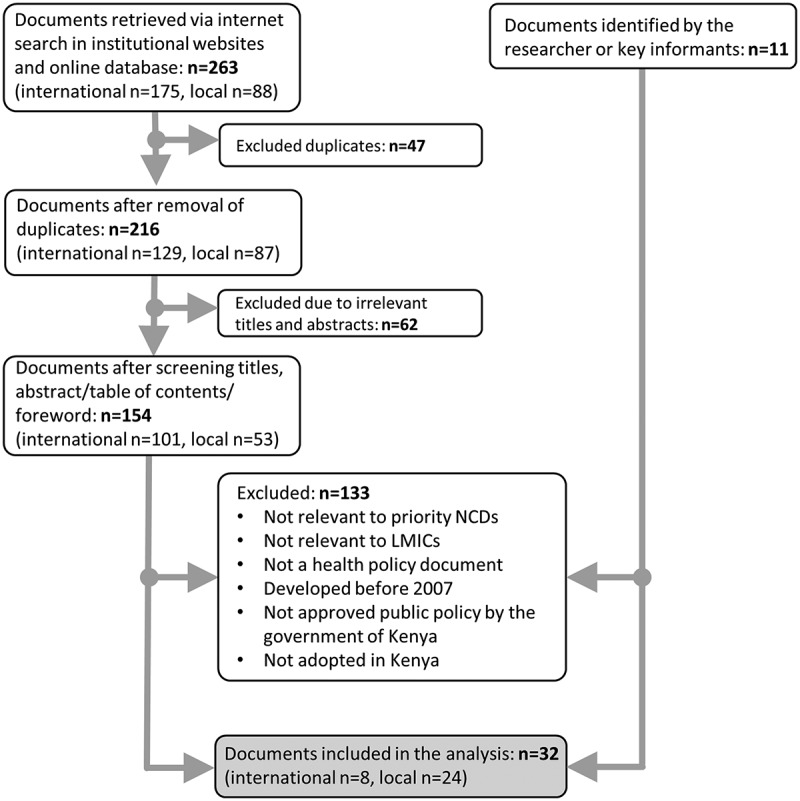
Flowchart showing the process of policy document collection and screening.

A search on PubMed, Google Scholar, UN websites, and government and diabetes association websites was performed. Search terms included [‘Diabetes’ OR ‘NCD’] AND [‘Policy’ OR ‘Strategies’ OR ‘Actions’ OR ‘Plan’ OR ‘Program’] AND [‘Kenya’]. Additionally, national policy documents were requested from the documents‘ authors and considered for analysis if confirmed to be relevant by the researcher, the documents’ authors and by key informants. A total of 32 diabetes-related policy documents was analyzed (Figure 1).

### Qualitative interviews

**Figure 2.**
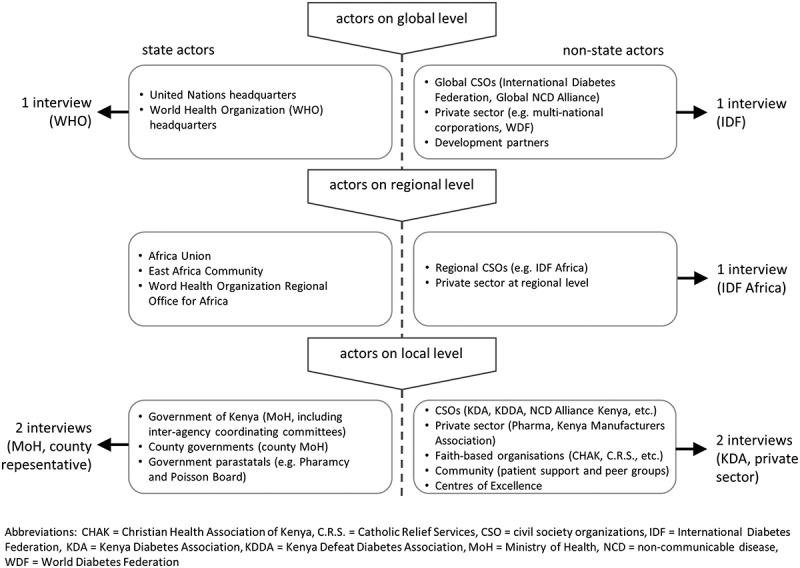
Multi-level stakeholders influencing diabetes policy processes in Kenya (2016).

Key informant interviews included experts from the main stakeholders in diabetes prevention and control in Kenya, regionally and globally. Purposive selection of informants was aided by stakeholder mapping (Figure 2). The informants were contacted via email, text messages and telephone and Skype calls. Confirmed interviews at each mapped level were conducted to explain the overall policy processes at that level. Similarly, within Kenya, additional interviews were conducted with the main diabetes policy stakeholders to reflect their views on the subject. Each interview was between 1 h up to 1 h and 30 min long. A topical guide was used to conduct the interviews, and it broke each session into themes such as:

- Background of the informant
- Status of policy development in Kenya or geographical area of interest including a ranking of the key players
- Status of implementation of policies for diabetes prevention and control
- Policy obstacles and gaps for implementation in diabetes prevention and control
- Solutions and suggested way forward (including where to put the emphasis on diabetes prevention and control)

After each section of the interview, key points were verbally summarized by the interviewer and further clarified by the interviewee.

### Data triangulation and analysis

All data were triangulated and augmented using a thematic analysis. The information was transcribed verbatim and analyzed using an inductive approach. Conceptual coding was performed and running themes identified. The similarity of responses was described, and exemplar opinions and ideas were quoted. The Walt and Gilson (1994) Policy Analysis triangle [32,33], a widely applied framework for health system analysis, was employed for further thematic analysis and to deconstruct the themes into an explanatory flow. This approach addresses complex interrelationships by separating policy analysis into context, actors, content, and process. Therefore, the contextual factors influencing policy and the policy background are addressed under context. Actors are stakeholders involved in the policy change. Content refers to what the policy mainly entails, whereas the process refers to conceptualization and application/implementation of the policy.

### Ethical considerations

Ethical approval for the study protocol was obtained from the authors’ institute and from the Kenyan MoH. Written informed consent was sought from key informants. Confidentiality and anonymity in the data processing was assured. The findings of the study were disseminated to the stakeholders who took part in the interviews.

## Results

The findings are presented following Walt and Gilson’s [32] domains of context, actors, content, and process.

### Context

The Kenyan health system for a long time was characterized by remnants of its post-colonial, three-tiered health system, including: (i) central government at the district, provincial and national levels, (ii) missionaries at sub-district levels, and (iii) local government in urban areas. Today the health system has been restructured into three sub-systems, e.g. (i) public sector (largest number of health-care facilities), (ii) commercial private sector, and (iii) faith-based organizations [4]. Since the re-introduction of user fees in 1989, out-of-pocket expenditure in Kenya remains high at 29% of total health expenditure (THE) and a corresponding 70% of domestic private health expenditure, highlighting a major barrier to healthcare access for Kenyans [34]. Government spending on health in 2014/15 was 4% of the national budget and 33.5% of THE [35], compared to the internationally recommended 15% of total government expenditure [36]. The remaining 37.5% of THE for the same year was directly from development partners and private sources. The adoption of a new constitution in 2010 ushered a devolved system of governance with 47 semi-autonomous counties. Healthcare received a 57% increase in total budget allocation between 2013 and 2015 [37] or a corresponding 45% (adjusted for inflation), largely due to increased allocations by administrative units besides the national government.

Prior to the UN declaration in 2011, Kenya already made steps towards reform for diabetes care. A first national diabetes program was launched in 2010 and operated under the MoH’s NCD department [38]. However, progress towards reversing the diabetes burden had been slow. An unstable political landscape, a global economic recession, and a fragmented national health system were some of the challenges noted by the MoH and civil society for diabetes. The then coalition government of two rival political parties split the MoH into two: one ministry for public health and another for medical services [39]. This resulted in duplicated roles and a constrained budget, which negatively impacted on national health functions including efforts towards fighting diabetes. Entities such as the National Diabetes Stakeholder Forum were also negatively impacted. The UN declaration in 2011 coincided with a constitutional change process in Kenya, during which diabetes civil society organizations and patient support groups responded to some of the challenges faced by leveraging citizens’ rights provisions in the new constitution to advance NCD policy discussions and rally the media to raise awareness [23]. The new constitution led to significant changes in Kenya’s political structure most notably the introduction of a devolved system of governance. One representative from the civil society noted:

‘[…] the devolution from the national government of health to counties has created new opportunities as well as new challenges because we have to now engage each particular county health ministry on its own and sometimes NCDs do not seem to be a priority to them [...]’

Under governmental devolution as described above, the responsibility for primary and secondary health services was decentralized from the national government under the MoH to 47 semi-autonomous county governments each with its own health ministry. The national MoH’s new role was to provide policy support and technical guidance for priority national programs. These policy changes are stipulated in the Kenya Health Policy 2012–2030 (now KHP 2014–2030) which includes ‘halt and reverse rising burden of NCDs’ as its second main policy objective, and calls for their subsequent inclusion in the Kenya Essential Package of Health [40]. This demonstrates increased prioritization of NCDs and successful local advocacy efforts in the post-UN declaration era. The KHP 2014–2030, based on the Constitution of Kenya 2010, the Kenya Vision 2030 development blueprint, and the end-term evaluation of the Kenya Health Policy framework 1994–2010 merged the two ministries back into one. Currently, the MoH’s five-year Kenya Health Sector Strategic Plans also direct policy for the health sector [40].

### Actors

The identified actors for diabetes policy development are mainly within the health sector. Key informants (Table 1) uniformly recognize the MoH’s mandate as the leading government agency for all matters pertaining to health and cite the importance of the MoH’s stewardship role. When the MoH recognizes that a disease is important, it convenes relevant stakeholders and seeks their insight and potential solutions. Together, a strategy to address the problem is developed. The policy negotiation process often goes according to plan, but occasionally, it is hindered by challenges ranging from antagonism among actors to factors beyond the health sector as described by key informants (Table 1, Table 2).

**Table 1.** Informants’ perceptions of the policy development process for diabetes/NCDs in Kenya.

Ministry of Health	‘The ministry takes the leadership but we involve all other partners in terms of we call them for a retreat or meeting then we discuss what are the priority areas – which areas do we need to cover to reverse the burden. We even involve the patients through an organization called the Kenya Defeat Diabetes Association which is the national umbrella for all the diabetes support groups.’
Donor in the Pharmaceutical Industry	‘It is very MoH driven. They involve us in development of guidelines. They do call us where they need our support.’
Civil Society	‘It (policy development) is inclusive within the health sector but on the other hand I feel it’s kind of exclusive. When you look at diabetes and other NCDs, you see that some of the other things that impact health, in terms of causative or predisposing factors to diabetes and other NCDs are beyond the health docket and yet we never involve these people.’
County	‘Before we used to receive the policy documents from the ministry and implement depending on the funds they send us. Now we receive the policies then develop our own depending on our priorities.’

**Table 2.** Perceptions on non-health sector involvement in policy development for diabetes and NCDs.

‘[…] NCDs in this country is something that is just gaining popularity it was not there before. Diabetes- as much as people are living with it and in the communities, they are suffering, most of the partners who are non-health partners do not see the need for them to be involved in diabetes for example let’s say finance, agriculture, trade, transport, security. They didn’t think that they can be involved in diabetes care or diabetes policy making so what we do is we give technical support then other NGOs that are dealing with diabetes come on board to give technical advice.’ (representative from MoH NCD department)
Referring to transport, agriculture, trade, urban planning, housing and security sectors: ‘So if we (the health sector) are talking about these things ourselves and not involving the other sectors – so that they are also sensitive to the issues around predisposing factors and put them into their development plans in the different sectors- then we are basically talking to ourselves. And I think we would be more effective when we involve them to create ownership at implementation level.’ (representative from IDF Africa)
‘We should involve non-health partners in everything so that we are not talking to them or directing them but they are part of the development of policy and implementation…because most of the times we are meeting them after we have developed the policies and we are now trying to negotiate. In my opinion we need to involve them earlier than later.’ (county health representative, Kenya)
‘Actually, the level of awareness outside the health sector and even within the health sector and among policy makers is still very low. These guys either they are closing their eyes or because there is no funding they just keep quiet but that is one of the challenges. We have low awareness among policy makers in terms of what needs to be done to reverse this trend or what needs to be done for prevention and also for care and treatment of the patient.’ (representative from diabetes civil society Kenya)
‘Players that contribute to NCD risk factors and those that contribute to prevention – their budgets are miles apart! This is clear even in our media. The advertisements that contribute to risk factors are more than those of prevention.’ (key informant from pharmaceutical sector)
‘We had tried to push the parliament to develop an NCD bill to become an act of parliament. Unfortunately, when it came back to the ministry some guys felt that we should have one act of parliament covering all NCDs instead of separately for each NCD. That proposed act of parliament was unfortunately shelved.’ (key informant from MoH-K)

The influence of civil society (including patient support groups) and the MoH’s leadership have been essential in leveraging efforts for diabetes prevention and control. However, contrary to UN declaration recommendations, the Kenyan diabetes policy processes remain largely health-sector driven (Table 2).

In addition to vested political and professional interests, a low level of awareness among policymakers towards diabetes features as a recurring challenge (Table 2). In response, the Kenyan MoH reinstated the National Diabetes Stakeholders Forum in 2016 to provide a platform for a more synergy-driven agenda. An inter-agency coordinating committee has also been created at the ministry, which interviewed stakeholders hoped will translate into a more multi-sectoral engagement for NCDs.

### Content

Analysis of policy documents developed prior to the 2011 UN declaration (1 January 2006 to 31 December 2011) and in the post-declaration period (1 January 2012 to 1 January 2016) demonstrated that all Kenyan diabetes-specific policies emerged before the UN declaration was passed. Retrieved policy documents largely reflect their alignment to African regional strategies developed in the buildup to the UN high-level meeting [41–43]. Policies currently feature diabetes under the NCDs umbrella or associated risk factors, which reflects a push to an integrated approach. In this regard, the Kenya Strategy on Prevention and Control of NCDs 2015–2020 represents the current overarching policy document for all NCDs in the country. The implementation of diabetes policies is currently mostly driven at the Kenyan national level. Technical meetings and awareness training by the MoH are underway at the county level to lobby for the inclusion of diabetes and NCDs into county health strategies. Counties which bear a proportionate burden of NCDs nationally are expected to prioritize this area of health policy.

Of the 31 analyzed policy documents (Table 3), two diabetes-related documents reflect changes in policy directions following the 2011 UN declaration. The Kenya National Diabetes Strategy (KNDS) 2010–2015 aims to prevent or delay the development of diabetes, improve quality of life and reduce complications and premature mortality [44]. The emphasis is on primary prevention of diabetes with interventions targeting obesity, physical inactivity, and unhealthy diet; resource mobilization; capacity building; partnership and coordination; diabetes policies, legislation and regulations; research; and monitoring and evaluation. The strategy was supported by accompanying guidelines for treatment and capacity building. The policy was context- and disease-specific and aimed to align with existing health programs. However, key respondents recognized the lack of a monitoring framework to evaluate the implementation progress.

**Table 3.** List of diabetes and/or NCD-related policies in Kenya.

policy document	level	lead agency/source ^a^	pre/post UN NCD declaration	considering NCDs	covering diabetes	covering NCD risk factors	prevention emphasis ^b^
United Nations Political Declaration on NCDs 2011	international	UN	(reference)	yes	yes	yes	p, s, t
Kenya Health Policy 2014–2030	national	MoH	post	yes	yes	yes	not defined
Kenya Health Sector Strategic Plan 2014–2018	national	MoH	post	yes	yes	yes	not defined
Kenya Vision 2030 Second Medium Term Plan 2013–2017	national	MoH	post	yes	no	yes	not defined
Kenya National Strategy for the Prevention and Control of NCDs 2015–2020	national	MoH	post	yes	yes	yes	p, s, t
Kenya National Nutrition Action Plan 2012–2017	national	MoPHS	post	yes	yes	yes	p
Kenya STEPwise Survey on NCD Risk Factors Report 2015	national		post	no	no	yes	p
Kenya Demographic Health Survey 2014	national	KnoBS	post	no	no	yes	not defined
Global Adult Tobacco Survey (ITC Policy Evaluation Report)	national		post	yes	no	yes	not defined
MoH Ministerial Investment Strategy 2014–2018	national/in-ministry	MoH	post	yes	yes	yes	p, s, t
Kenya National Health Accounts fiscal years 2012/2013, 2014/2015	national	MoH	post	yes	no	no	not defined
Kenya National Diabetes Strategy 2010–2015	national	MoPHS	pre	yes	yes	yes	p
Kenya National Clinical Guidelines for Management of Diabetes Mellitus July 2010	national	MoPHS	pre	yes	yes	yes	p, s, t
Kenya Diabetes Comprehensive Care Manual July 2010	national	MoPHS	pre	yes	yes	yes	p, s, t
Kenya National Diabetes Educators Manual July 2010	national	MoPHS	pre	yes	yes	yes	p, s, t
National Food and Nutrition Security Policy 2011	national	GoK	pre	yes	yes	yes	p
Kenya national Physical Activity Action Plan	national	MoPHS	post	yes		yes	p
Kenya Health Policy 1994–2012	national	MoH	pre	no	no	yes	not defined
Kenya National School Health Strategy 2010–2015	national	MoPHS/MoE	pre	yes	no	yes	p
Kenya Vision 2030 First Medium-Term Plan 2008–2012	national	GoK	pre	no	no	yes	not defined
2008–2013; 2013–2020 Action Plan for the Global Strategy for the Prevention and Control ofnon-communicable Diseases	international	WHO	pre, post	yes	yes	yes	p, s, t
WHO Framework Convention on Tobacco Control	international	WHO FCTC	pre	yes	yes	yes	p
Global strategy on diet, physical activity and health	international	WHO	pre	yes	yes	yes	p
Global Strategy to reduce the harmful use of alcohol	international	WHO	pre	yes	yes	yes	p
Moscow Declaration of the First Global Ministerial Conference on Healthy Lifestyles andnon-communicable Disease Control	international	WHO	pre	yes	yes	yes	p, s, t
Ministerial declaration high level segment of the Economic and Social Council, 2009	international	WHO	pre	yes	yes	yes	not defined
Brazzaville Declaration on NCD Prevention and Control in WHO AFRO Region	regional	WHO AFRO	pre	yes	yes	yes	p, s, t
Libreville Declaration on Health and Environment in Africa, August 2008	regional	WHO AFRO	pre	yes	yes	yes	p, s, t
NCD Statement of the Commonwealth Heads of Government, November 2009	international		pre	yes	yes	yes	p, s, t
IDF Diabetes Strategy for sub-Saharan Africa	regional	IDF Africa	pre	yes	yes	yes	p, s, t
Diabetes Strategy for WHO Africa Region 2007	regional	WHO AFRO	pre	yes	yes	yes	p, s, t

^a^ UN: United Nations, MoH: Ministry of Health, MoPHS: Ministry of Public Health and Sanitation, GoK: Government of Kenya, MoE: Ministry of Education, WHO: World Health Organization, WHO FCTC: WHO Framework Convention on Tobacco Control, ITC: International Tobacco Control, WHO AFRO: WHO African region, IDF Africa: International Diabetes Federation Africa

^b^ p: primary, s: secondary, t: tertiary

The Kenya National Strategy for Prevention and Control of NCDs 2015–2020 was developed in the post-2011 declaration era and aligned with WHO’s Global NCD Action Plan 2013–2020 and accompanying Global Monitoring Framework as well as the objectives of the Brazzaville declaration on NCDs [6,43]. The Kenyan document emphasizes an integrated approach to address NCDs and attendant risk factors and advocates for integrating NCDs into existing primary health-care platforms. In contrast to KNDS 2010–2015, it includes a set of national NCD targets and an implementation monitoring framework. However, key informants mentioned the lack of concrete baseline data as a limitation. Considering local realities, some judged the Kenyan national targets as too close to the ambitious international targets.

### Process: challenges and promising strategies

Funding, monitoring, and evaluation of policy interventions remain some major challenges for diabetes control in Kenya. Policy implementation was described as ‘piecemeal’ and with predominantly weak monitoring and evaluation systems. The country relies on donor-led or international research to develop policies, which often do not mirror local problems. For example, according to the Kenya Diabetes Management Information Centre, diabetes among Kenyan youth is a growing problem that is not accorded sufficient attention in existing policies. One respondent from the pharmaceutical sector notes:

‘We do not have a lot of studies coming out locally that we could conclusively say will be able to guide us on how we are able to approach treatment, etc.. Therefore, we have to borrow heavily from international frameworks and especially when it comes to best practices. If you look at our own manuals and presentations that we use to do our trainings, they are very heavily borrowed from international policies. Apart from nutrition maybe which I will say is heavily localized.’

Kenya has registered progress on diabetes through policy interventions in various sectors (Table 4). The National Hospital Insurance Fund, a government body mandated to facilitate Kenya’s universal health coverage agenda, includes diabetes care in its medical cover package. Patient empowerment is evident through the involvement of patient associations in policy development. As confirmed by key informants, the MoH inter-agency coordination committee on NCDs recently included players such as the manufacturing sector. The MoH proposed tax incentives to foods and beverages to encourage the production and marketing of healthy products. However, taxation policies for population risk factor modulation for NCDs and their implementation are generally reported as a complex challenge. For example, implementation of the 2007 Tobacco Act regulations did not begin until 2014. The establishment of centers of excellence for diabetes management and care, and capacity building for in-service and pre-service human resource (Table 4) remain essential for secondary and tertiary prevention efforts across the country.

**Table 4.** Summary of policy interventions for diabetes prevention and control in Kenya.

field	prevention type	target groups, institutions	measures	responsibility/executers	status in June 2016
education, advocacy, and empowerment	primary	children in schools	integrate nutrition in school curriculum physical exercises offering healthy meals healthy school policies	national government (MoH, MoE, Teachers‘ Service Commission, etc.) county governments	physical education and nutrition education is integrated into the school curricula school health policies are currently being implemented in some parts of Kenya
	primary	workplaces, employers and employees	information of workers on the role of physical activity encouragement and facilitation of the implementation of work-related healthy lifestyle practices	all public and private sectors	implementation and monitoring at the national level are difficult the public sector has no policy private sector companies and cooperatives have in-house policies private and NGO sectors sponsor national walks, e.g. at the World Diabetes Day, and World Health Day.
	primary	public/com-munities/high risk groups and indivi-duals, health facilities and public campaigns	advocacy, distribution of IEC materials on healthy lifestyle habits usage of social and mainstream media	MoH at national and county level partners: health-care workers, religious leaders, community leaders, media entities	IEC materials are available in all public health facilities and diabetes clinics according to informants (no means of verification) media campaigns happen during World Diabetes Day the rest of the year campaigns is too expensive with less effect according to MoH the Kenya Defeat Diabetes Foundation formed as a MoH-led initiative 300 patient support groups with 40,000 members across the counties
	secondary	diabetes patients, the public, health facilities/hospitals	patient education and empowerment IEC materials self-management training	patient support groups MoH and partners: health-care workers, civil society, media entities	country wide capacity building trainings by MoH and partners taking place in health-care facilities and schools, etc. diabetes clinics established in all sub-counties across Kenya
	primary secondary tertiary	schools, communities, hospitals	capacity building of health-care workers (in-service and pre-service for medical schools) IEC materials implementation of care, treatment and management guidelines	MoH civil society patient support groups	diabetes centers of excellence present in Machakos, Kakamega, Mombasa 1,300 health-care workers trained on diabetes foot care, management of diabetes wounds and ulcers.
food policy	primary	population-wide	food regulations (to include fortification with micronutrients, limiting salt contents, food labeling, advertising) incentives for food manufacturers to replace unhealthy ingredients with healthy ones trans-fats regulations agricultural policies towards the production of traditional/indigenous food varieties food price policies to make healthy food more affordable	MoH nutrition department agriculture sector manufacturing and processing industries	food and nutrition policy developed but implementation status currently unknown high taxation in the agriculture sector has discouraged local production of fresh food leading to increased purchasing in urban areas positive: counties are now encouraging farming and agriculture as a development agenda the Kenya Manufacturers Association is now part of the MoH Inter-Agency Coordinating Committee for NCDs
tobacco and alcohol policies	primary	population-wide	implementation and scaling up of the Tobacco Act 2007 and of the recommendations of the Global Adult Tobacco Survey/International Framework Convention on Tobacco Control implementation of alcohol regulations (including minimum drinking age, banning of illicit alcohol)	county council authorities nationwide national authority for the Campaign Against Alcohol substance and Drug abuse the Kenyan police (for enforcement)	Tobacco Act developed in 2007 but its regulations not implemented until 2014; currently in the process of implementation Global Adult Tobacco Survey conducted, report disseminated. smoking zones developed and smoking in common areas fully outlawed Alcohol Act fully implemented illicit brews fully outlawed.
urban design and transportation policy	primary	population-wide	inter-ministry collaboration in urban planning development	inter-ministry collaboration city and county council authorities	so far, no inter-ministerial policy for city planning relevant ministries not involved in the policy development.
medical interventions	secondary, tertiary	patients with diabetes and high risk groups	reduction of the price of insulin increase the supply of insulin and essential medicines and testing kits optimization of the value chain adherence to treatment care and management guidelines	MoH pharmaceutical industries Kenya Revenue Authority	‘Base of the Pyramid’ project for increased insulin access by subsidizing costs (initiative of the pharmaceutical industry and the government of Kenya) ‘Change Diabetes’ project for diabetes prevention and control among children and young people
screening	primary: high risk groups, secondary: blood sugar monitoring	population-wide	routine blood glucose test in patient triaging Malaria testing combined with a blood glucose screening	health-care workers self-management for patients with diabetes health-care facilities (all levels)	glucose screening integrated into malaria testing for children in some counties
treatment and prophylaxis	primary: IGT/high risk groups, secondary/tertiary: diabetes patients	high risk indi-viduals (with IGT) and dia-betes patients	glucose monitoring adherence to treatment care and management guidelines self-monitoring/self- management	patients (self-management), health-care workers MoH (capacity building)	The National Hospital Insurance Fund has included coverage of diabetes patients in its outpatient care package

Abbreviations: MoH: Ministry of Health; MoE: Ministry of Education; IEC: information, education, and communication; IGT: impaired glucose tolerance; NGO: non-governmental organization.

## Discussion

This study has demonstrated Kenya’s positive steps towards reform of diabetes prevention and control measures. The inclusion of patients with diabetes in various levels of policy development processes has been important in shaping national interventions against NCDs. Notably, these have translated into successful advocacy and lobbying efforts by civil society and patient support groups within the national discourse. However, while early milestones in the Kenyan policy development for diabetes prior to and following the 2011 UN NCD declaration were important, they have not been sufficient so far to achieve global targets since persistent contextual challenges such as in the political landscape, funding, human resources, etc., undermined such efforts.

In Kenya, as in many LMICs, the 2011 UN NCD declaration fostered political will and action and raised awareness at a policy level thus leveraging more opportunities for collaboration among stakeholders. The promulgation of a new Kenyan constitution created a positive legal framework, e.g. for the diabetes community to integrate existing efforts into the wider NCD agenda. These ‘policy windows’ in Kenya elicited ‘priority bursts’ for NCDs and diabetes which, as described by Shiffman and Smith [45], ascribe to theoretical affirmations of the four factors that precede the generation of global political priority for a global health issue: actor power, ideas, political contexts and issue characteristics/features of the problem [45].

Implementation of policies for diabetes, and more generally NCDs, in Kenya, is piecemeal, curative-focused and up until the policy period 2010–2015, characterized by weak monitoring frameworks. Contrary to the recommendations of the 2011 UN declaration on NCDs, efforts in Kenya are largely health-sector driven and do not have sufficient political power to have a population-wide impact on NCD morbidity. Stewardship of the national MoH remains important considering the devolved governance structure and heterogeneous actor landscape. However, this context also provides opportunities for more targeted interventions. The step from KNDS 2010–2015 to the Kenyan National Strategy for Prevention and Control of NCDs 2015–2020 illustrates a move away from a disease-specific approach to a broader one. Ownership of NCD policy processes could be further strengthened by multi-sector engagement beyond the health sector such as from fiscal policies to urban planning. This would require political responsibility at higher levels. Mendis and Beaglehole et.al [46,47] emphasize the importance of policymakers acknowledging how public policies have bearing on behavioral NCD risk factors outside the health sector and environmental risk factors [46,47]. The limited power and fewer opportunities for influencing politics of those involved in prevention efforts compared to those who contribute to NCD risk factors reveal an important dynamic that could impede population risk-factor modulation.

Currently, numerous opportunities exist within the devolved system of governance in Kenya to extend prevention at a population level and in particular, to align efforts to policy windows within the country’s universal health coverage and sustainable development agenda. The framework of the sustainable development goals to which Kenya subscribed could provide a further opportunity to address overarching issues (e.g. poverty) as NCD drivers while maintaining improvement strategies within health systems. Frameworks that promote policy coherence, such as WHO’s health-in-all-policies, could also help to sustain synergy within complex political interrelationships [48].

Existing policies remain strongly aligned to international documents. However, while diabetes and NCDs featured frequently in analyzed high-level policy documents, locally generated evidence based on locally available data when translating global declarations into local policy development processes remained scant. This was also evident in the challenges of monitoring local policy interventions against set international targets. This problem of data generation is not unique to Kenya and has been mirrored in other African countries [49–52] and to a greater extent LMICs. Investment in implementation research to generate relevant local data and improve progress monitoring is warranted for evidence-informed decision-making.

Unwin et al. [53] claimed a research agenda on NCDs already in 2001, encompassing data on the level, coverage, and quality of health care [53]. Research on factors such as health beliefs (including the perceptions and attitudes of health-care providers) and the social and economic well-being of communities, but also on the structures and processes within the health system is needed. Additionally, research on policy implementation and evaluation of interventions regarding the effective and efficient use of resources are lacking. This appeal remains relevant in many aspects until today. The Lancet NCDs and Injuries Poverty Commission could provide a forum to further address this agenda and prove relevant for policy development. Juma et al. [52] push for the generation of local evidence on multi-sectoral action to inform policy development in Africa [52], as has been successfully done in high-income countries. This approach would heed WHO’s call for a ‘whole-of government’ approach in addressing NCDs in LMICs.

However, there are further lessons to be learned from the Kenyan case for LMICs and we suggest applying a general system theory perspective to accelerate NCD strategy implementation in LMICs. Although most health systems are not comparable directly due to country-specific structural and political characteristics, some elements are similar across the globe, as for instance the organization in tiers with primary care at the base and increasing specialization towards upper levels. In general, decision-making for the advancement of a health system and the successful implementation of services top-down requires the bottom-up aggregation of valid high-quality data at grassroots, even in a ‘whole-of-government’ approach. As can be seen in Kenya, political will and passing strategies and policies aligned with international guidelines alone, even with the participation of concerned patient groups, does not necessarily shape the health systems at the ground accordingly. Implementing new strategies requires a comprehensive approach, with a given flexible framework on the ministerial level and data generation on the ground key to realize tailored solutions and feedback for informed decision-making. However, if this holds true in general, implementation research should develop a general modular system kit of transferable elements proven to be necessary for the successful implementation of NCD services (prevention and control) in all LMICs (instead of complete single-country solutions), accompanied by country-specific detailed components. In particular, more basic effective tool kits for NCD data generation and processing at the grassroots level are needed to enable monitoring of a process-oriented implementation.

## Conclusion

The health sector in Kenya already included the emerging issue of diabetes prevention and control prior to the UN declaration on NCDs in 2011 in its health strategic planning, considering the input of the civil society and patient support groups. However, there is an implementation gap between the policies passed and the reality of diabetes prevention and control. The derived strategies are based on scant local evidence and accompanied by weak monitoring systems. Additionally, the non-health sector so far is hardly involved. In consequence, Kenya is far from meeting the global diabetes targets. To achieve population-wide impact, Kenya needs to strengthen the ownership of NCD policy processes by multi-sector engagement such as from fiscal policies to urban planning. Kenya’s sustainable development agenda provides an opportunity to back diabetes prevention and control from the top political level, to address cross-sectoral issues and to enhance political power to fight general diabetes risk factors. Furthermore, local data generation should be engineered in order to develop tailored prevention and control measures and to enhance feedback to the political levels to enable evidence-based decision-making.

## Paper context

NCDs are an important health problem in Kenya. We present Kenya’s challenges and milestones in translating international NCD declarations in response to diabetes through policy reforms. Civil society groups seized a window of political opportunity lobbying successfully for changes in policy development for NCD and diabetes locally. However, some policies disregard local realities, implementation monitoring is neglected. Multi-sector engagement is needed to address cross-sectoral issues and to enhance political power to fight diabetes risk factors.
